# Glucose Metabolism and Its Complicated Relationship with Tumor Growth and Perfusion in Head and Neck Squamous Cell Carcinoma

**DOI:** 10.1371/journal.pone.0166236

**Published:** 2016-11-08

**Authors:** Noriyuki Fujima, Tomohiro Sakashita, Akihiro Homma, Kenji Hirata, Tohru Shiga, Kohsuke Kudo, Hiroki Shirato

**Affiliations:** 1 Department of Diagnostic and Interventional Radiology, Hokkaido University Hospital, Sapporo, Japan; 2 Department of Otolaryngology-Head and Neck Surgery, Hokkaido University Graduate School of Medicine, Sapporo, Japan; 3 Department of Nuclear Medicine, Hokkaido University Graduate School of Medicine, Sapporo, Japan; 4 Department of Radiation Medicine, Hokkaido University Graduate School of Medicine, Sapporo, Japan; 5 The Global Station for Quantum Medical Science and Engineering, Global Institution for collaborative research and education, Sapporo, Japan; Ludwig-Maximilians-Universitat Munchen, GERMANY

## Abstract

**Objective:**

To determine the relationship between tumor glucose metabolism and tumor blood flow (TBF) in head and neck squamous cell carcinoma (HNSCC).

**Methods:**

We retrospectively analyzed 57 HNSCC patients. Tumor glucose metabolism was assessed by maximum and mean standardized uptake values (SUVmax and SUVmean) obtained by ^18^F-fluorodeoxyglucose positron-emission tomography. TBF values were obtained by arterial spin labeling with 3-tesla MRI. The correlations between both SUVs and TBF were assessed in the total series and among patients divided by T-stage (T1–T3 and T4 groups) and tumor location (pharynx/oral cavity and sinonasal cavity groups). Pearson’s correlation coefficients were calculated for significant correlations.

**Results:**

Significant correlations were detected: a negative correlation in the advanced T-stage group (TBF and SUV max: r, −0.61, SUVmean: r, −0.62), a positive correlation in the non-advanced T-stage pharynx/oral cavity group (TBF and SUVmax: r, 0.70, SUVmean: r, 0.73), a negative correlation in the advanced T-stage pharynx/oral cavity group (TBF and SUVmax: r, −0.62, SUVmean: r, −0.65), and a negative correlation in the advanced T-stage sinonasal cavity group (TBF and SUVmax: r, −0.61, SUVmean: r, −0.65).

**Conclusion:**

Significant correlations between glucose uptake and TBF in HNSCC were revealed by the division of T-stage and tumor location.

## Introduction

For head and neck (HN) squamous cell carcinomas (SCCs), several studies have demonstrated that tumor glucose metabolism and tumor blood flow (TBF) are important biological factors for the diagnosis and treatment, respectively [1–4]. Positron-emission tomography (PET) with ^18^F-fluorodeoxyglucose (FDG) depicts the tumor metabolic rate of glucose. The tumor metabolic rate is one of the important factors that reflect tumor aggressiveness, and thus the determination of a tumor’s FDG uptake has been reported to improve the accuracy of the diagnosis, staging, and detection of recurrence in HNSCCs [1, 2]. The TBF has also been reported to reflect tumor characteristics such as tumor neo-angiogenesis, and has thus been found to be useful for the pretreatment assessments of patients' need for chemoradiotherapy, and for therapeutic monitoring [3, 4]. In addition, the recent use of the arterial spin labeling (ASL) technique has allowed the noninvasive measurement of the tissue blood flow even in head and neck lesions [5–7]. With the ASL technique, we have been able to measure TBF more easily due to its noninvasiveness compared to the dynamic contrast-enhanced technique.

The relationship between tumor glucose metabolism and TBF has been unclear, and a very limited number of studies have investigated this relationship [8–12]. Hirasawa et al. [9] described an inverse correlation between the tumor standardized uptake value (SUV) and TBF parameters in patients with HN tumors. However, Bisdas et al. reported a significant positive linear correlation between SUV and TBF values in HNSCCs [10]. Other reports indicated that there was no correlation between SUV and TBF values in HNSCC patients [10–12]. These conflicting results are difficult to explain. Several tumor factors such as T-staging and different primary sites may have contributed to the differing findings.

The aim of the present study was to investigate the correlation between tumor glucose metabolism and TBF in patients with HNSCC, examining the division of tumor location and the T-stages of the primary sites.

## Materials and Methods

### Patients

This retrospective study was approved by our institutional review board (approved by Hokkaido University Hospital, ID; 014–0230), and informed consent was waived. We retrospectively evaluated the cases of 57 patients with HNSCC with the following inclusion criteria: (1) the patient was first diagnosed (not a recurrent case) histopathologically as having HNSCC, (2) both MR and PET/CT scanning was performed before any treatment, and (3) the scan interval between MR and PET/CT was <3 weeks. The patients were 50 males (mean age 61.2 yrs, range 33–80 yrs) and seven females (mean age 64.6 yrs, range 51–89 yrs). The primary lesions of the 57 patients and the T stages were as follows: the sinonasal cavity in 28 patients (T2 in one patient, T3 in 13, T4a in 10, and T4b in four) and the pharynx or oral cavity in 29 patients (T1 in one, T2 in eight, T3 in six, T4a in 12, and T4b in two). The patients with a pharynx SCC included the oropharynx and hypopharynx only; there was no nasopharynx SCC.

### FDG-PET imaging technique

FDG-PET/CT images were acquired with a Biograph 64 PET/CT scanner (Asahi-Siemens Medical Technologies, Tokyo, Japan). The patients fasted for ≥6 h before the injection of FDG (4.5 MBq/kg) and waited 60 min post-injection. The energy window was 425–650 keV. The transaxial and axial fields of view (FOVs) were 58.5 cm and 21.6 cm, respectively. A 3-min emission scanning in 3D mode was performed for each bed position. Attenuation was corrected with X-CT images acquired without contrast media. The images were reconstructed with an iterative method integrated with a point spread function (TrueX) [13]. Fully 3D PET reconstruction with a system matrix derived from point source measurements was conducted [12]. The reconstructed image had a spatial resolution of 8.4 mm full width at half maximum (FWHM) and a matrix size of 168×168 with a voxel size of 4.1×4.1×2.0 mm. The SUV was defined as the tissue concentration of radioactivity (kBq/mL) divided by the injected dose per body weight (kBq/g), as is commonly used [14].

### ASL imaging technique

#### Imaging parameters

The pseudo-continuous ASL (pCASL) technique was used in all ASL imaging. The acquisition of pCASL was performed by using multi-shot spin-echo echo-planar imaging to obtain control and labeled images. The labeling slab was placed just under the bifurcation of the internal and external carotid arteries by using the coronal T2-weighted image (T2WI) as the reference. The pCASL parameters were as follows: labeling duration, 1650 ms; post-label delay, 1280 ms; TR, 3619 ms; TE, 18 ms; flip angle, 90°; number of shots, two; FOV, 230×230 mm; matrix, 80×80; slice thickness, 5 mm; number of slices, 15; acceleration factor for parallel imaging, two; and scanning time, 5 min 11 s.

#### TBF quantification by pCASL

We calculated the TBF of the pCASL (*f*) from the signal difference (ΔM), which was calculated by subtracting the labeled image from the control image, using the previously described equation:

$$f=\frac{\Delta M\lambda R_{1a}\exp(\omega R_{1a})}{2M_{0}\alpha }{\left[1-\exp(-\gamma R_{1a})\right]}^{\text{-}1}$$ (1)

where R_1a_ is the longitudinal relaxation rate of the blood (0.67 s^−1^), γ is the labeling time (1.65 s), ω is the post-labeling delay time (1.28 s), α is the labeling efficiency (0.85), and λ is the blood/tumor-tissue water partition coefficient (1.0 g/mL) [7]. M_0_ is the equilibrium magnetization of the tumor tissue, which was estimated from the signal intensity of the control image and the tumor longitudinal relaxation rate obtained with the T1 map. Using Eq (1), we created the TBF maps on a pixel-by-pixel basis. We used mathematical software (MATLAB ver. 2012a, MathWorks, Natick, MA) to calculate the TBF values.

Other conventional MR images were obtained with the following parameters: (a) axial T1-weighted images (T1WI) with a spin-echo sequence (TR 450 ms, TE 10 ms, FOV 240×240 mm, 512×512 matrix, slice thickness 5 mm, inter-slice gap 30%, and (b) axial and coronal T2WI with a turbo spin-echo (TSE) sequence with fat suppression (Axial images: TR 4500 ms, TE 70 ms, TSE factor 9, FOV 240×240 mm, 512×512 matrix, slice thickness 5 mm, inter-slice gap 30%. Coronal images: TR 4500 ms, TE 70 ms, TSE factor 9, FOV 240×240 mm, matrix 512×512, slice thickness 4 mm, inter-slice gap 30%).

### Data analysis

#### The calculation of the mean and maximum SUVs

For the semiquantitative evaluation of the FDG uptake in the primary tumor, we estimated the uptake in the tumor using the mean and maximum SUVs (SUVmean and SUVmax). We measured the SUVmax and SUVmean for each tumor by determining the automated region of interest (ROI) using an isocontour threshold method. A threshold SUV value of 2.5 was set for the tumor ROI delineation to exclude the central necrosis or nearly normal tissue uptake [15]. The highest SUV value within the tumor ROI was considered the SUVmax, and the average of the SUV values within the tumor ROI was considered the SUVmean. If a tumor extended to two or more slices, the highest SUV value in all slices tumor was defined as the SUVmax, and the mean SUV value of all pixels in all ROIs of the tumor was calculated as the SUVmean.

#### The calculation of TBF values

Each patient’s primary tumor was outlined by a board-certified neuroradiologist with 19 yrs of experience (A.T.). The delineation was performed on the axial T2WI with a polygonal ROI, and the ROI was then copied onto a TBF map. The ROI delineation was performed using Image J software (U.S. National Institutes of Health, Bethesda, MD) to include the soft-tissue mass (and to exclude the normal or inflammatory tissue) in the ROI obtained from the T2WI imaging findings. T1WI was also used as a guide to determine the ROI. To avoid vascular artifacts in the ROI, we also delineated the area of the vessel signal void on the T2WI, and this area was excluded from the TBF measurement. Any strong high-signal area with T2WI that suggested necrosis was also excluded. The pCASL TBF value for each patient was determined as the mean of the TBF values in the delineated ROI. If the tumor extended into two or more slices on the TBF map, the mean TBF of all pixels in all ROIs of the tumor was calculated as the TBF value [16].

### Statistical analysis

We analyzed the correlations between the SUV values (max and mean) and TBF values using Pearson’s correlation coefficients (r < 0.2, poor correlation; r = 0.2–0.4, weak correlation; r = 0.41–0.6, moderate correlation; r = 0.61–0.8, good correlation; r ≥ 0.81, excellent correlation). The correlation analysis between SUV and TBF values was performed for the (1) all-patients group, (2) each patient group divided by tumor location (the pharynx/oral cavity group and the sinonasal cavity group), and (3) each patient group divided by tumor T-stage (non-advanced group: T1–T3 and advanced group: T4a/T4b). As a subgroup analysis, a correlation analysis was performed in patient groups with more detailed division: (a) the pharynx/oral cavity group with the non-advanced (T1–T3) patient group, (b) the pharynx/oral cavity group with the advanced (T4) patient group, (c) the sinonasal cavity group with the non-advanced (T1–T3) patient group, and (d) the sinonasal cavity group with the advanced (T4) patient group. The level of significance was set at p<0.05.

## Results

The averages of the TBF, SUVmax and SUVmean values in each patient group are summarized in Table 1.

**Table 1:** The SUVmax, SUVmean and TBF values of the 57 HNSCC patients.

Group division	SUVmax	SUVmean	TBF (ml/100g/min)
All patients (n = 57)	18.3±7.2	9.2±3.4	124.8±40.1
Division of tumor location			
All SCC patients with pharynx/oral cavity (n = 29)	16.6±7.3	8.4±3.5	115.9±37.7
All SCC patients with sinonasal cavity (n = 28)	19.9±6.9	9.9±3.6	133.9±41.0
Division of tumor T-stage			
All SCC patients with non-advanced T-stage (T1-3) (n = 29)	17.7±7.9	8.9±3.8	132.3±42.2
All SCC patients with advanced T-stage (T4) (n = 28)	18.8±6.5	9.4±2.9	116.9±36.8
Each subgroup divided by tumor location and T-stage			
Non-advanced T-stage (T1-3) with pharynx/oral cavity (n = 15)	14.6±6.5	7.4±3.3	116.1±41.8
Advanced T-stage (T4) with pharynx/oral cavity (n = 14)	18.9±7.8	9.5±3.5	115.6±34.3
Non-advanced T-stage (T1-3) with sinonasal cavity (n = 14)	21.0±8.3	10.5±3.7	149.6±36.4
Advanced T-stage (T4) with sinonasal cavity (n = 14)	18.8±5.2	9.5±2.3	118.2±40.4

Footnote: Data are mean ± SD. SCC: squamous cell carcinoma, SUVmax: maximum standardized uptake value, SUVmean; mean standardized uptake value, TBF: tumor blood flow.

In the overall patient analysis, no significant correlation was observed between the SUVmax or SUVmean and TBF values (p = 0.74, 0.61).

In the analysis divided based on the tumor location in all patients, no significant correlation was observed in either the pharynx/oral cavity or sinonasal cavity groups (p = 0.39, 0.57 respectively).

**Fig 1:**
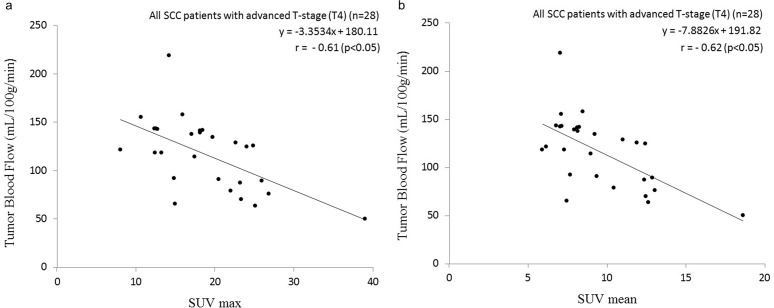
Scatterplot of the SUV and TBF values in all patients with an advanced T-stage (T4) SCC. There were significant negative correlations between the SUVmax and TBF and between the SUVmean and TBF values. Pearson’s correlation coefficient between SUVmax and TBF was −0.61 (p<0.05) (a), and that between SUVmean and TBF was −0.62 (p<0.05) (b).

The analysis of the groups divided based on the tumor T-stage in all patients revealed a tendency for a moderate positive correlation between the SUVmax (r = 0.41, p = 0.09) or the SUVmean (r = 0.43, p = 0.15) and the TBF values in non-advanced T-stages (T1–T3), but no significance was observed. In contrast, there was a significant negative correlation between the SUV (both the SUVmean and SUVmax) and the TBF values in the advanced T-stage (T4) patients (SUV max: r = −0.61, p<0.05; SUVmean: r = −0.62, p<0.05) (Fig 1).

**Fig 2:**
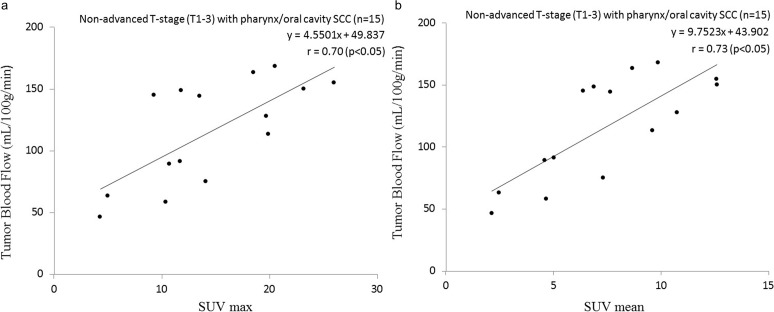
Scatterplot of the SUV and TBF values in the pharynx/oral cavity group with non-advanced T-stage (T1–T3) tumors. In the subgroup analysis of the pharynx/oral cavity group with non-advanced T-stage (T1–T3), significant positive correlations were observed between the SUVmax and TBF and between the SUVmean and TBF values. Pearson’s correlation coefficient between the SUVmax and TBF was 0.70 (p<0.05) (a), and that between the SUVmean and TBF was 0.73 (p<0.05) (b).

In the subgroup analysis, there was a significant positive correlation between the SUV (both the SUVmax and SUVmean) and TBF values in the subgroup analysis of the pharynx/oral cavity group with non-advanced T-stage (T1-3) SCCs (SUVmax: r = 0.70, p<0.05, SUVmean: r = 0.73, p<0.05) (Fig 2).

**Fig 3:**
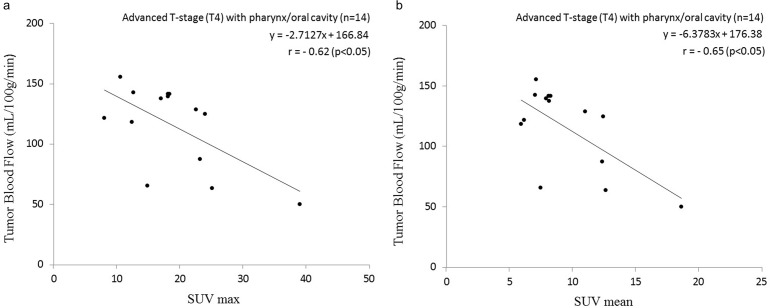
Scatterplot of the SUV and TBF values in the pharynx/oral cavity group with advanced T-stage (T4) tumors. Significant negative correlations were observed between the SUVmax and TBF and between the SUVmean and TBF values. Pearson’s correlation coefficient between SUVmax and TBF was −0.62 (p<0.05) (a), and that between SUVmean and TBF was −0.65 (p<0.05) (b).

In addition, there was a significant negative correlation between the SUV (both SUVmax and SUVmean) and TBF values in the subgroup analysis of the pharynx/oral cavity group with advanced T-stage (T4) tumors (SUVmax: r = −0.62, p<0.05, SUVmean: r = −0.65, p<0.05) (Fig 3).

**Fig 4:**
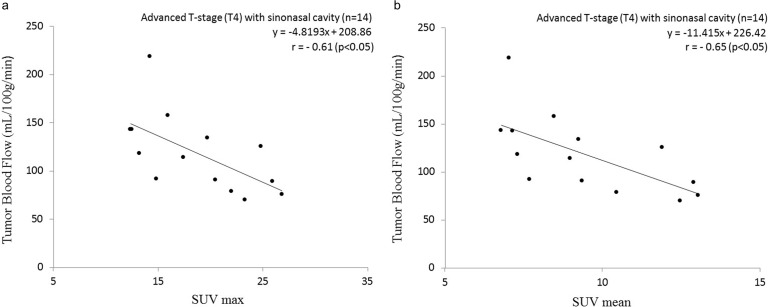
Scatterplot of the SUV and TBF values in the sinonasal cavity group with advanced T-stage (T4) tumors. Significant negative correlations were observed between the SUVmax and TBF and between the SUVmean and TBF values. Pearson’s correlation coefficient between SUVmax and TBF was −0.61 (p<0.05) (a), and that between SUVmean and TBF was −0.65 (p<0.05) (b).

In the subgroup analysis of the sinonasal cavity group with non-advanced T-stage (T1-3) SCCs, there was no significant correlation between the SUV and TBF values (r = −0.18, p = 0.79). However, significant negative correlations were observed between the SUV (both the SUVmax and SUVmean) and the TBF values in the sinonasal cavity group with advanced T-stage (T4) tumors (SUVmax: r = −0.61, p<0.05, SUVmean: r = −0.65, p<0.05) (Fig 4).

## Discussion

Our present findings revealed the positive correlation of SUV max/mean and TBF values in the non-advanced T-stage pharynx/oral cavity group, and a negative correlation of SUV max/mean and TBF values in the advanced T-stage patients in both the pharynx/oral cavity and sinonasal cavity groups. In contrast, no correlation was found in the total patient analysis.

Bisdas et al. observed a positive correlation between SUV uptake and TBF values [9], as we did in our non-advanced T-stage patients with pharynx/oral cavity SCCs (shown in Fig 2A and 2B). In the Bisdas study, only two patients with a T-stage of T4 were present in the total population of patients (n = 15). The patient characteristics related to T-stage could be considered to be similar between patients in the Bisdas study and those of non-advanced T-stage group in our present study. The neovascularity and angiogenesis are thought to develop during the period of SCCs’ progression from the early stage, and the tumor glucose uptake increases in parallel. Such a correlation was detected in our study population. However, there was no correlation in our patient group of sinonasal cavity SCCs. The reason for this is unclear and difficult to explain. In our sinonasal cavity group, there was no T1-stage patient and only one T2-stage patient. In sinonasal cavity cancer cases, it is often difficult to detect the primary lesion at the early stage (T1 or T2) compared to pharynx cancers. Our number of patients (n = 1) with very early stage (T1 or T2) SCCs, which are expected to have both low TBF and SUV uptake, might be inadequate to identify a correlation. The difference in the determination of the T-stage also contributed to this result; the determination of the T1–T3 pharynx/oral cavity cancers was based on the tumor size, whereas the T-stages of the sinonasal cavity cancers were determined based on their local invasiveness around the sinonasal cavity.

In contrast, Hirasawa et al. reported a negative correlation of SUV and TBF values in patients with head and neck tumors, although not all of the histopathological diagnoses were SCC (they examined a total 16 tumors including nine SCCs) [8]. Our present analyses revealed a negative correlation of SUV and TBF values in patients with advanced T-stage status regardless of their primary lesions (see Figs 1A, 1B, 3A, 3B, 4A and 4B). Although the detailed data of the patients' T-stages in the Hirasawa study were not presented and remain unclear, a negative correlation between the TBF and SUV values was obtained only in their patient group with large tumor sizes (>8 cm). We therefore suspect that that large-tumor group might have included mostly advanced T-stage patients; it would resemble the advanced T-stage group in our present study. Hirasawa et al. concluded that the negative correlation was due to the inclusion of tumors with uncoupling of the TBF and SUV uptake, and they speculated that the reason for this uncoupling was that angiogenesis cannot maintain an adequate level of blood supply as the tumor grows and will result in relatively low TBF, whereas the tumor itself will remain aggressive with a high SUV in anaerobic glycolysis. They also noted that such cases of uncoupling might be related to tumor hypoxia because of the low tissue oxygenation caused by low TBF. Another study also reported that tumor tissues in a hypoxic condition were related to low TBF [17]; low TBF in hypoxic tumor tissues might lead to the mismatch of TBF and SUV uptake. Additionally, Lin et al. stated that the percentage of tumors with a positive hypoxic marker (i.e., erythropoietin receptor expression) tended to be higher in T3–4 tumors compared to T1–2 tumors [18]. The cases of advanced T-stage patients can include a number of HNSCCs with tumor hypoxia, additionally, hypoxic tissue might be related to the TBF-SUV mismatch as mentioned above; these factors can lead to the negative correlation between TBF and SUV in advanced T-stage group. Goh et al. demonstrated that a lower ratio of TBF/SUV was related to hypoxia-inducible factor 1 expression [19]. Our identification of several patients with the combination of low TBF and high SUV (i.e., a low TBF/SUV ratio) corresponds to Goh et al.’s finding and might have a certain degree of relation to tumor hypoxia, although the type of cancer in the Goh et al. study (colorectal cancer) differed from that examined in our present investigation.

Three other research groups reported that they found no correlation between SUV uptake and the TBF [10–12]. We suspect that this is because the study populations of those studies included a number of both non-advanced and advanced T-stage patients: 12 T4-stage patients among a total of 35 patients [10], five T4-stage patients among a total of 15 patients [11], and five T4-stage patients among a total of 22 patients [12]. Since both positive and negative correlations were present in the overall correlation assessments in those studies, a significant correlation may not have been detected.

Bases on these results, we speculate that the different conclusions regarding the relationship between SUV and TBF in past reports are due mainly to the difference in the T-stage of the primary tumors in the study populations. In addition, different locations of the primary tumor might have some influence. We speculate that at the early stage, primary HNSCCs will develop with parallel increases in their glucose uptake and TBF, whereas when primary SCCs progress to a more advanced stage such as T4, some of the tumors will show a mismatch of TBF and SUV (low TBF and high SUV), and these tumors with TBF-SUV uncoupling will cause the negative correlation of TBF and SUV.

This study has several limitations. First, the SUV and TBF values were not acquired at the same time. For several patients, there was an approx. 3-week interval between the two scans. A slight change of SUV or TBF values may be observed at an interval of several weeks. Second, more detailed subgroup analyses such as those of tumor differentiation or human papillomavirus (HPV) status were not performed. In such analyses, larger numbers of patient data would be necessary. Further analysis is required to investigate and reveal the tumor biological correlations. In addition, our speculation related to the two-parameter mismatch of TBF and SUV for the determination of tumor hypoxia was not confirmed by a gold standard method such as a PET study with a specific tracer (^18^F-fluoroazomycin arabinoside (FAZA) PET or ^18^F-fluoromisonidazole (FMISO) PET [20]) or histological staining (e.g., with pimonidazole). Further investigations are needed to address this limitation.

In conclusion, there was a significant correlation between glucose uptake and TBF in HNSCCs based on the division of T-stage and tumor location. This result can clarify some of the tumor characteristics of HNSCCs, and it provides useful information for the diagnosis, treatment and care of HNSCC patients in clinical settings.
